# Evaluating large language models using the Type 2 Diabetes Health Education guideline: a comparative analysis of ChatGPT-4.1, Claude-4.0, DeepSeek-V3, and ERNIE Bot 4.5 Turbo

**DOI:** 10.3389/fpubh.2026.1900281

**Published:** 2026-08-19

**Authors:** Zhaoxia Huang, Yuxin Bai, Junyue Luo, Zihang Zhang, Chunlan Hu, Xinyu Hu, Liying Ruan, Jiaxin Zeng, Qianhui Jia, Luyao Deng, Meifeng Xiong, Ying Zhou, Ziyi Qi, Wenxi Lyu, Xu Li

**Affiliations:** 1 Department of Ophthalmology, The Affiliated Hospital of Southwest Medical University, Luzhou, China; 2 School of Nursing, Southwest Medical University, Luzhou, China; 3 College of Literature, Yancheng Teachers University, Yancheng, China

**Keywords:** health information quality, large language models, patient education, readability, type 2 diabetes mellitus

## Abstract

**Objective:**

To systematically evaluate the quality and readability of health information generated by four large language models (LLMs) in response to inquiries regarding type 2 diabetes mellitus (T2DM), using an authoritative Chinese clinical guideline as the reference standard.

**Methods:**

A total of 124 standardized questions were extracted from the Chinese Type 2 Diabetes Popular Science Guidelines. Six endocrinologists and diabetes specialists conducted independent, blind evaluations using the CLEAR tool (Completeness, Lack of false Information, Evidence, Appropriateness, Relevance) and PEMAT-P (Patient Education Materials Assessment Tool for Printable materials). Response characteristics were also recorded. Between-model differences were tested using the Kruskal–Wallis H test with Bonferroni pairwise comparisons.

**Results:**

All four models achieved total CLEAR scores within the “very good” range (19–25), with no significant differences seen between models (χ^2^ = 1.985, *p* = 0.576). No significant differences were observed in the dimensions of Lack of false information (χ^2^ = 7.644, *p* = 0.054), Evidence (χ^2^ = 2.309, *p* = 0.511), and Relevance (χ^2^ = 7.516, *p* = 0.057). However, significant differences emerged in Completeness (χ^2^ = 47.661, *p* < 0.001) and Appropriateness (χ^2^ = 88.360, *p* < 0.001). Claude-4.0 received the lowest score in Completeness (median 4.00, IQR 3.00–5.00) but achieved the highest ranking in Appropriateness (median 4.00, IQR 4.00–5.00). On the PEMAT-P, understandability differed significantly across models (χ^2^ = 159.120, *p* < 0.001), yet all models surpassed the 70% threshold, with ChatGPT-4.1 highest (median 91.91%, IQR 91.91–100.00%). However, despite significant differences among the various models (χ^2^ = 354.023, *p* < 0.001), only ERNIE Bot 4.5 Turbo (median 75.00%, IQR75.00–75.00%) surpassed the 70% threshold, with no single model demonstrating consistent superiority across all dimensions.

**Conclusion:**

Although the four LLMs generally provide accurate and pertinent information regarding type 2 diabetes, enduring limits in actionability and inconsistencies among models in content completeness and understandability restrict their effective use in diabetic patient education. Future development should prioritize stronger step-by-step behavioral guidance and differentiated, scenario-specific model deployment to enhance their value in patient-facing diabetes self-management support.

## 1 Introduction

Type 2 diabetes mellitus (T2DM) represents one of the most formidable public health challenges of the twenty-first century, with its global prevalence having risen dramatically over recent decades (1). Not only does it pose a severe threat to patients’ life, health, and quality of life, but it also imposes a heavy economic and service burden on healthcare systems worldwide (2). More noteworthy is that T2DM, as a chronic disease that requires lifelong management, its clinical control effectiveness largely depends on the patient’s own disease cognition level and self-management ability, and this process highly relies on reliable and timely health information support (3).

The rapid development of Internet technology and widespread use of mobile intelligent devices are catalyzing a significant digital shift in the acquisition of health information. A national survey of American adults indicates that over 80% of participants pursue health-related information online (4), and this trend is also reflected globally. Patients today get medical information through a growing array of digital means, including websites, social media platforms, and artificial intelligence chatbots (5). Large Language Models (LLMs) may produce coherent, human-like text responses to natural language inquiries, facilitating quicker and more accessible health information retrieval for individuals. Substantial evidence indicates significant differences in the quality, reliability, and comprehensibility of information conveyed through different channels. However, many studies have shown that the medical information provided by LLMs still has obvious deficiencies in the dimensions of content accuracy, information traceability transparency and language readability, and the consistency with clinical guidelines is also limited (6–8). Low-quality or misleading health information might significantly endanger patient comprehension and negatively influence medical decisions (9–11). Consequently, it is necessary to systematically evaluate the health information generated by LLMs to preserve public health.

Previous studies have shown that LLMs have the potential to deliver medical information. Certain models even exceed conventional web resources or human experts on knowledge quality and empathy in specific tasks (12, 13). However, their performance is not stable, and there are still concerns about the accuracy, reliability and potential bias of their answers (14, 15). In recent years, although the research on LLM in different medical specialties has gradually increased (16, 17), in the specific field of type 2 diabetes, the use of standardized and validated evaluation tools to compare and evaluate multiple LLMs is still scarce. For example, certain studies only evaluated the quality of ChatGPT’s answers to diabetes related questions (18, 19), lacking a cross model horizontal comparison perspective. It is worth noting that ChatGPT series models (especially ChatGPT-4) are relatively outstanding in the medical and health information question and answer task in the clinical scene of breast cancer, the scene of generating educational materials for patients undergoing cardiac catheterization, and even in the clinical chemistry examination, and their comprehensive quality is better than other models in the same period (16, 20, 21). However, most of the above conclusions are based on a single model comparison. Based on the above research gap, this study chose to systematically compare and evaluate the response quality of mainstream LLM on T2DM related issues. This study employs the Chatgpt-4.1 model, given its superior comprehensive capabilities demonstrated in numerous studies. Additionally, it includes Claude 4.0, a leading international model, alongside domestic representatives DeepSeek-V3 and ERNIE Bot 4.5 turbo, totaling four LLMs for comparative analysis. This selection aims to enhance research support for the application of large language models in the provision of diabetes health information.

## 2 Materials and methods

### 2.1 Study design

**Figure 1:**
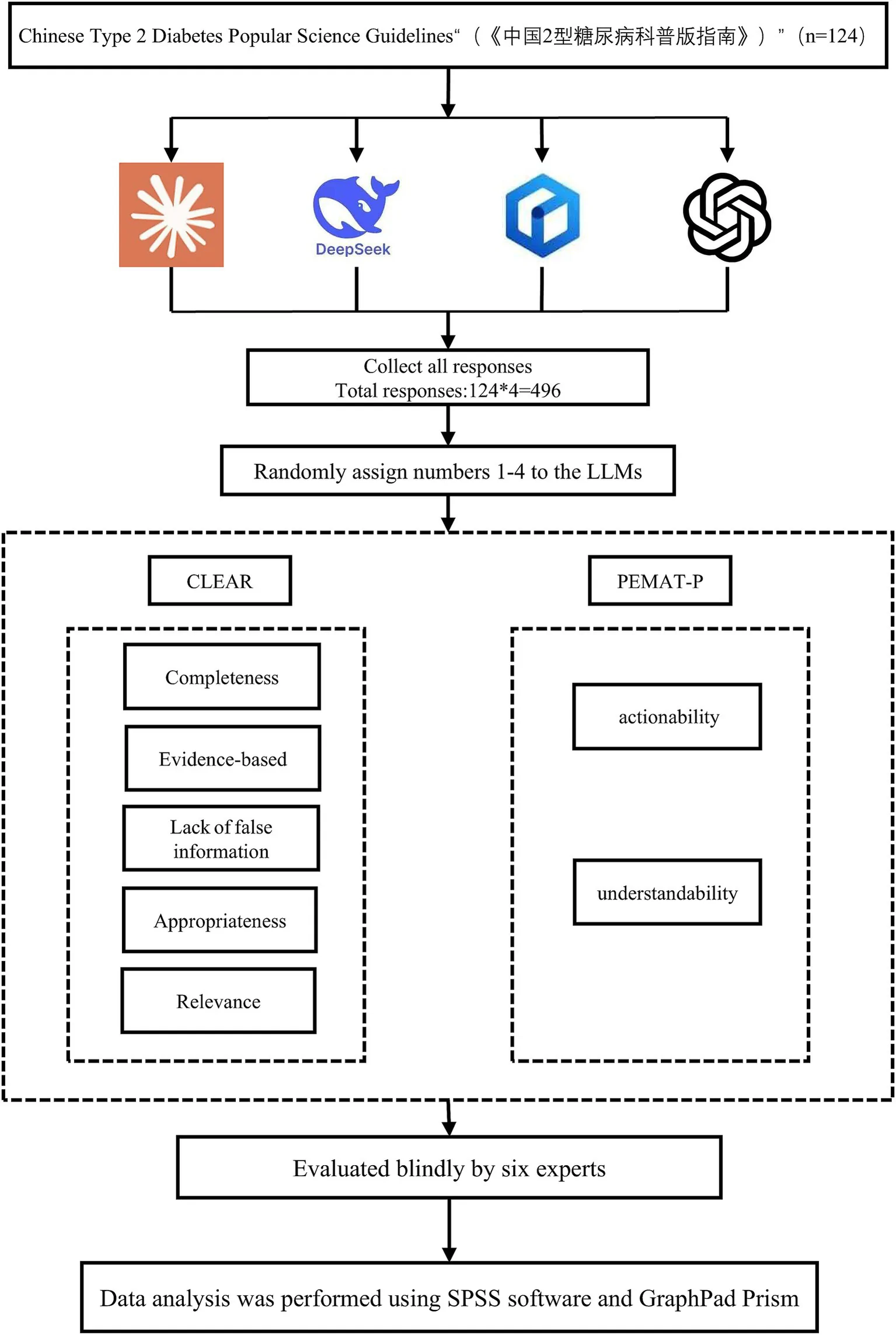
Flowchart of overall study design.

This study aims to systematically assess and compare the quality of health information generated by four large language models (LLMs) in the field of type 2 diabetes management. We adopted a cross-sectional approach and selected PEMAT-A/V and CLEAR as assessment tools to comprehensively evaluate the performance of Claude-4.0 (https://claude.ai), ChatGPT-4.1 (https://chat.openai.com), DeepSeek-V3 (https://chat.deepseek.com), and ERNIE Bot 4.5 Turbo LLMs (https://yiyan.baidu.com/). Our research is based on the Chatbot Assessment Reporting Tool (CHART) statement for chatbot health advice studies (22). This study presented 124 questions to four large language models, derived from a reputable professional publication on type 2 diabetes. On July 23, 2025, Researcher A and Researcher B independently submitted all 124 questions from the guideline book to each LLM-Chatbot. After four LLMs generate responses, Researcher C uniformly organizes and eliminates AI characteristics for expert scoring. Additionally, we recorded the fundamental response characteristics of each AI model, including total response time (in seconds), character count (in characters), time to First byte (in seconds), and character generation rate (characters/s). The responses are compiled in a Microsoft Excel file. An expert evaluation panel was convened to conduct independent, blinded scoring of all AI-generated responses. The panel comprised six board-certified endocrinologists and diabetes specialists, each possessing a minimum of 10 years of continuous clinical experience in diabetes diagnosis, management, and patient education. The AI-generated responses were subsequently subjected to independent scoring by the six panelists. The resultant scores were statistically analyzed using IBM SPSS Statistics 31.0. and GraphPad Prism 10.6.0 to assess inter-rater reliability and overall response quality (Figure 1).

### 2.2 Data source

All questions were obtained from the book titled “Chinese Type 2 Diabetes Popular Science Guidelines” (《中国2型糖尿病科普版指南》), a comprehensive guidance book that closely integrates the characteristics of the Chinese population and provides scientific guidance for the clinical diagnosis of T2DM in China (23).

The “Chinese Type 2 Diabetes Popular Science Guidelines” adopts a question-and-answer format to deliver standardized diabetes education information to patients, using accessible, easy-to-understand language supplemented by extensive illustrations and tables. The guideline also systematically addresses and clarifies common misconceptions surrounding the prevention and management of diabetes. The book consists of fourteen chapters, including the fundamental knowledge of diabetes, dietary management, physical activity, pharmacological therapy, self-monitoring, prevention and management of complications, self-management strategies, weight management, emerging technologies, and diabetes management in special populations. To ensure comprehensive coverage of the common clinical questions and foundational knowledge about type 2 diabetes, all questions and their corresponding answers contained in this guideline were selected as the question set and reference standard for the present study. Each of the 124 questions was submitted independently to each LLM chatbot using a newly initiated conversation session. A new chat window was created for every question to prevent the models from accessing previous conversation contexts. No follow-up questions, additional prompts, or conversational interactions were provided after the initial question. All questions were entered using the same standardized format across the four LLM platforms to ensure consistency of data collection. Total response time and Time to first byte were measured using a browser, which more closely approximates the response speed as perceived by users in actual product use. To ensure the reproducibility of measurements across trials, the following measures were implemented during testing: the same device and the same version of the Chrome browser were used throughout; the browser cache was cleared and all other tabs and browser extensions were closed prior to testing, in order to minimize interference from background processes on page rendering; and a stable network environment was maintained during testing, with other high-bandwidth network activities avoided concurrently. Time to first byte was defined as the time interval from the execution of the “Send” action until the first byte of response data was received by the browser. Total response time was defined as the interval from the execution of the “Send” action until the completion of response data transmission. Character count was calculated using the LEN function in Microsoft Excel, with all visible characters in the complete generated response, including letters, numbers, punctuation marks, and spaces, being counted. Character generation rate (characters/s) was calculated by dividing the character count by the corresponding response time. A blinded evaluation approach was employed in this study, in which researchers assigned anonymous labels to the four models, designated as Model 1, Model 2, Model 3, and Model 4, respectively (16, 24). Furthermore, all content that could potentially reveal the identity of the source model was removed from the responses before evaluation, thereby ensuring that expert evaluators remained fully blinded to the origin of each response throughout the assessment process. Also, we removed markdown formatting from the AI-generated responses while preserving text, numbers, punctuation, spaces, line breaks, and tab characters. To ensure consistency and comparability of measurements across models, identical standardization procedures were applied to responses from all four AI systems in this study.

### 2.3 Evaluation methodology and procedure

A panel of six endocrinologists and diabetes specialists was recruited to form an expert evaluation committee, The inclusion criteria were as follows: (1) A minimum of 10 years of clinical experience in diabetes management; (2) Proficiency in pharmacology, therapeutic interventions, lifestyle management for diabetes, and knowledge of diabetes-related complications; (3) A professional title of Associate Chief Physician or above. Inter-rater reliability among the six experts was assessed using a two-way random-effects model with absolute agreement (ICC(2,k)), and an ICC value ≥0.75 was considered acceptable.

We systematically evaluated Claude-4.0, ChatGPT-4.1, DeepSeek-V3, and ERNIE Bot 4.5 Turbo using the CLEAR tool and PEMAT-A/V assessment tool, with the “Chinese Type 2 Diabetes Popular Science Guidelines” serving as the reference standard (25, 26).

To assess the quality of health information delivered by AI-based models, we selected the CLEAR scale to assess five themes: completeness of content, Lack of false information in the content, Evidence supporting the content, Appropriateness of the content, and Relevance. The CLEAR scale adopts a 5-point Likert scale, with each item scored as follows: “excellent” = 5, “very good” = 4, “good” = 3, “satisfactory/fair” = 2, or “poor” = 1. The range of CLEAR scores was 5–25, divided arbitrarily into three categories: 5–11 categorized as “poor” content, 12–18 categorized as “average” content, and 19–25 categorized as “very good” content. Detailed scoring criteria are provided in Table 1.

**Table 1:** Scoring criteria of the CLEAR tool (each domain rated from 1 to 5).

Grade	Definition
Completeness: Is the content sufficient?	
5	-The answer is highly comprehensive and in-depth, omitting no key content. The content is balanced and thorough, with sufficient and profound explanations.
4	-The answer is comprehensive and has adequate depth, covering the most relevant aspects and providing sufficiently detailed content.
3	-The answer contains the necessary core information with limited depth but includes the key aspects of relevant health issues.
2	-The answer provides basic information, but there are obvious information gaps. Key information is incomplete, which may lead to partial misunderstanding.
1	-The answer is severely incomplete; most necessary information is missing, the information provided is minimal, and key content is lacking, which may lead to serious misdiagnosis or health risks. The information is extremely one-sided, omitting multiple key elements.
Lake of false knowledge: Is the content accurate?	
5	-The answer is entirely accurate, without any errors or misleading information. Data and factual statements are precise. The provided information is trustworthy.
4	-The answer is accurate in most aspects, highly consistent with current medical consensus and guidelines, with only minor errors or inaccuracies. The advice is reliable and based on scientific evidence, and the information sources are trustworthy.
3	-The answer is generally accurate, but there are some minor errors. Most of the content is consistent with current medical consensus.
2	-The answer contains several obvious medical errors or misleading statements, with some key information inconsistent with current medical evidence.
1	-The answer contains multiple serious medical errors or false information that may lead to dangerous self-diagnosis or treatment decisions. The advice provided contradicts existing medical consensus or guidelines. Erroneous information may result in serious health consequences or a delay in seeking medical care.
Evidence: Is the content evidence-based?	
5	-Supported by substantial evidence, the latest scientific findings, and fully verified conclusions.
4	-Most information is supported by sufficient scientific evidence, citing reliable and relatively recent sources. The content is consistent with current evidence-based medical practice, with almost no obvious bias or misleading content.
3	-Most core information has some scientific evidence to support it. Cited sources are generally reliable but limited in depth and breadth.
2	-Only a tiny amount of scientific evidence supports the provided information.
1	-There is no evidence to prove it. All the information is incorrect.
Appropriateness: Is the content clear, concise, and easy to understand?	
5	-The information is easy to understand without ambiguity. All users can easily understand the information.
4	-Most of the information is clear. Generally free from ambiguity, well-organized, and presented in logical order. Ordinary users can easily understand the information.
3	-The information has basic explanations, but it may not be comprehensive enough. The content is concise, and ordinary users can understand most of it, but some clarification may be needed.
2	-The information contains numerous unexplained medical terms, some of the content is redundant or insufficiently detailed, and it even has confusing logic. Ordinary users must make additional efforts to understand it, such as consulting reference materials.
1	-The content contains technical terms that ordinary people find difficult to understand. Sentences are unclear and ambiguous, potentially misleading users.
Relevance: Is the content free from irrelevant information?	
5	-The answer addresses the medical query directly, with high relevance to all aspects of the medical question. It may also provide additional information that is highly pertinent to the medical context.
4	-The answer is relatively targeted, with a few aspects irrelevant to the medical issue. It also provides some other information.
3	-The answer is relevant but cannot completely solve the problem.
2	-The answer has some relevance to the medical query but may not cover all medical aspects, or it may contain some extraneous information. It generally responds to the medical query, but could be more focused.
1	-The answer lacks relevance to the medical query. It may be off-topic, medically inaccurate, or provide information that does not pertain to the medical question.

Based on the alignment between our research objectives and the tool characteristics, we adopted the PEMAT-P for printable materials (understandability: 17 items; actionability: 7 items) to evaluate these constructions. Each item was independently assessed by the six experts as “Agree” (1 point), “Disagree” (0 points), or “Not Applicable (N/A)” according to the PEMAT-P manual. Items marked as “Not Applicable” were excluded from the calculation. The understandability and actionability scores were calculated by dividing the total number of “Agree” responses by the total number of applicable items and multiplying the result by 100%. PEMAT-P score (%) = (Total Agree points / Total applicable items) × 100. According to the PEMAT-P developers, scores ≥70% indicate that the educational material is considered understandable or actionable, whereas scores <70% indicate that further improvement is needed. The PEMAT-P framework is particularly suited for assessing patient education materials, as it systematically evaluates whether information is presented in a manner that patients can comprehend and whether it provides clear, actionable guidance for health behaviors. PEMAT-P additionally assesses whether educational materials provide clear behavioral guidance and actionable recommendations, making it particularly suitable for evaluating AI-generated patient education materials. The evaluation tool has been widely adopted to evaluate patient education materials generated by artificial intelligence (AI) models (27, 28). Detailed scoring criteria can be found in Table 2.

**Table 2:** Patient education materials assessment tool for print materials (PEMAT-P) scoring standard.

Item	Description
Section 1: Understandability	
1	-The material makes its purpose completely evident
2	-The material does not include information or content that distracts from its purpose
3 4	-The material uses common, everyday language -Medical terms are used only to familiarize audience with the terms. When used, medical terms are defined.
5	-The material uses the active voice
6	-Numbers appearing in the material are clear and easy to understand
7	-The material does not expect the user to perform calculations
8	-The material breaks or “chunks” information into short sections
9	-The material’s sections have informative headers
10	-The material presents information in a logical sequence
11	-The material provides a summary
12	-The material uses visual cues (e.g., arrows, boxes, bullets, bold, larger font, highlighting) to draw attention to key points
15	-The material uses visual aids whenever they could make content more easily understood (e.g., illustration of healthy portion size)
16	-The material’s visual aids reinforce rather than distract from the content
17	-The material’s visual aids have clear titles or captions
18	-The material uses illustrations and photographs that are clear and uncluttered
19	-The material uses simple tables with short and clear row and column headings
Section 2: Actionability	
20	-The material clearly identifies at least one action the user can take
21	-The material addresses the user directly when describing actions
22	-The material breaks down any action into manageable, explicit steps
23	-The material provides a tangible tool (e.g., menu planners, checklists) whenever it could help the user take action
24	-The material provides simple instructions or examples of how to perform calculations
25	-The material explains how to use the charts, graphs, tables, or diagrams to take actions
26	-The material uses visual aids whenever they could make it easier to act on the instructions

Each item is rated as ‘Yes’ (1), ‘No’ (0), or ‘Not Applicable (NA)’. A ‘Yes’ is given when 80–100% of the material meets the criterion. The final score is: (Total score/maximum possible score) × 100.

We selected CLEAR and PEMAT rather than other commonly used assessment tools, such as the Global Quality Score (GQS) or DISCERN, based on several methodological reasons. CLEAR and PEMAT provide complementary frameworks specifically designed for patient education materials: CLEAR captures content quality dimensions (completeness of content, Lack of false information in the content, Evidence supporting the content, Appropriateness of the content, and Relevance), while PEMAT assesses understandability and actionability—making them ideally suited for our research objectives. GQS primarily functions as an online webpage evaluation tool, designed to assess website quality, making it unsuitable for evaluating AI-generated text that lacks the structural elements inherent to web-based content (29, 30). In contrast, although DISCERN is a validated tool for assessing health information quality, it was primarily developed for evaluating written materials focusing on treatment choices and the quality of information sources, making it unsuitable for evaluating AI-generated text (31, 32). Many AI-generated responses in this study covered a broader range of diabetes education topics, including disease knowledge, lifestyle management, self-monitoring, complication prevention, and special populations, rather than only treatment decision-making. Therefore, DISCERN may not comprehensively capture the multidimensional characteristics of LLM-generated patient education content. Accordingly, CLEAR was selected as a more suitable instrument for evaluating the informational quality of AI-generated diabetes education materials, while PEMAT was used to assess understandability and actionability. We integrated all AI model responses and scoring criteria into a questionnaire instrument, which was distributed to expert evaluators. Experts conducted scoring through questionnaires. For each question-model pair, the six expert ratings were averaged to generate a single aggregated score. These question-level aggregated scores were then used as the unit of analysis for statistical comparisons, resulting in four model-specific datasets containing 124 observations each.

### 2.4 Statistical analysis

Data analysis was conducted using IBM SPSS Statistics 31.0, and graphical presentations were generated using GraphPad Prism 10.6.0. Statistical results were presented as mean, standard deviation (SD), median, and interquartile range (IQR). Normality was assessed using the Kolmogorov–Smirnov test. Since the data were not normally distributed, the Friedman test was used to compare differences among the four LLMs. When significant differences were detected, Bonferroni-adjusted pairwise comparisons were performed to identify specific differences between individual models. All statistical tests were two-sided, and a *p* value <0.05 was considered statistically significant.

### 2.5 Ethical considerations

This study analyzed responses from 4 large language models regarding T2DM. Since the study did not involve patients directly, ethics committee approval was not required. All model outputs were collected via their respective official interfaces.

## 3 Results

### 3.1 Response analysis of 4 large language models

Table 3 presents the basic response characteristics of large language models (LLMs) as chatbots across 124 questions. The Friedman test revealed significant differences in response time among the four LLMs (χ^2^ = 339.842, *p* < 0.001, Kendall’s W = 0.914), time to first character (χ^2^ = 300.465, *p* < 0.001, Kendall’s W = 0.808), character count (χ^2^ = 348.774, *p* < 0.001, Kendall’s W = 0.938), and character generation rate (χ^2^ = 352.539, *p* < 0.001, Kendall’s W = 0.948).

**Table 3:** Basic response characteristics of large language models.

Variable	Descriptive statistics	Claude-4.0 (*N* = 124)	DeepSeek-V3 (N = 124)	ERNIE Bot 4.5 Turbo(N = 124)	ChatGPT-4.1 (N = 124)	χ^2^	Kendall’s W	*p* value
Response time	Mean ± SD	14.63 ± 2.21	36.63 ± 6.18	28.56 ± 5.56	10.73 ± 3.87	339.842	0.914	<0.001
	Median (IQR)	14.12 (12.96–16.29)	36.41 (32.79–39.98)	28.02 (24.56–32.65)	9.72 (8.04–12.52)			
Time to first byte	Mean ± SD	4.65 ± 0.91	4.26 ± 0.92	1.38 ± 0.35	1.37 ± 0.51	300.465	0.808	<0.001
	Median (IQR)	4.43 (4.12–4.92)	4.08 (3.77–4.53)	1.28 (1.20–1.48)	1.23 (1.11–1.43)			
Character count	Mean ± SD	498.27 ± 92.54	906.41 ± 167.02	1292.52 ± 213.97	713.10 ± 170.24	348.774	0.938	<0.001
	Median (IQR)	478.50 (436.25–544.50)	905.50 (789.25–1008.00)	1277.50 (1162.00–1431.25)	679.00 (571.50–811.25)			
Character generation rate	Mean ± SD	34.16 ± 4.19	24.80 ± 2.37	45.69 ± 4.14	70.79 ± 17.90	352.539	0.948	<0.001
	Median (IQR)	34.63 (30.95–36.86)	24.60 (23.51–26.21)	45.52 (42.81–48.43)	68.06 (59.52–82.93)			

N, simple size, SD, standard deviation, IQR, interquartile range, χ^2^, Friedman chi-square statistic, Kendall’s W, Kendall’s coefficient of concordance (effect size for the Friedman test), Kendall’s W values of 0.10, 0.30, and 0.50 were considered small, moderate, and large effects, respectively.

In terms of response time, ChatGPT-4.1 was the shortest, followed by Claude-4.0 and ERNIE Bot 4.5 Turbo. DeepSeek-V3 had the longest response time. For the time to first character, ChatGPT-4.1 and ERNIE Bot 4.5 Turbo exhibited comparable time to first byte values, while Claude-4.0 demonstrated the longest time to first character. In character count, Claude-4.0 provided the most concise responses, and ERNIE Bot 4.5 Turbo generated the longest responses. Character generation rate also showed significant differences. ChatGPT-4.1 achieved the highest generation rate, while DeepSeek-V3 achieved the lowest. The effect size analysis demonstrated very large differences in response characteristics among the four LLMs. Kendall’s W values ranged from 0.808 to 0.948, indicating very large effect sizes for response time, time to first character, character count, and character generation rate. These findings suggest that the evaluated models differed substantially in response efficiency and output characteristics.

### 3.2 Content quality assessment

**Figure 2:**
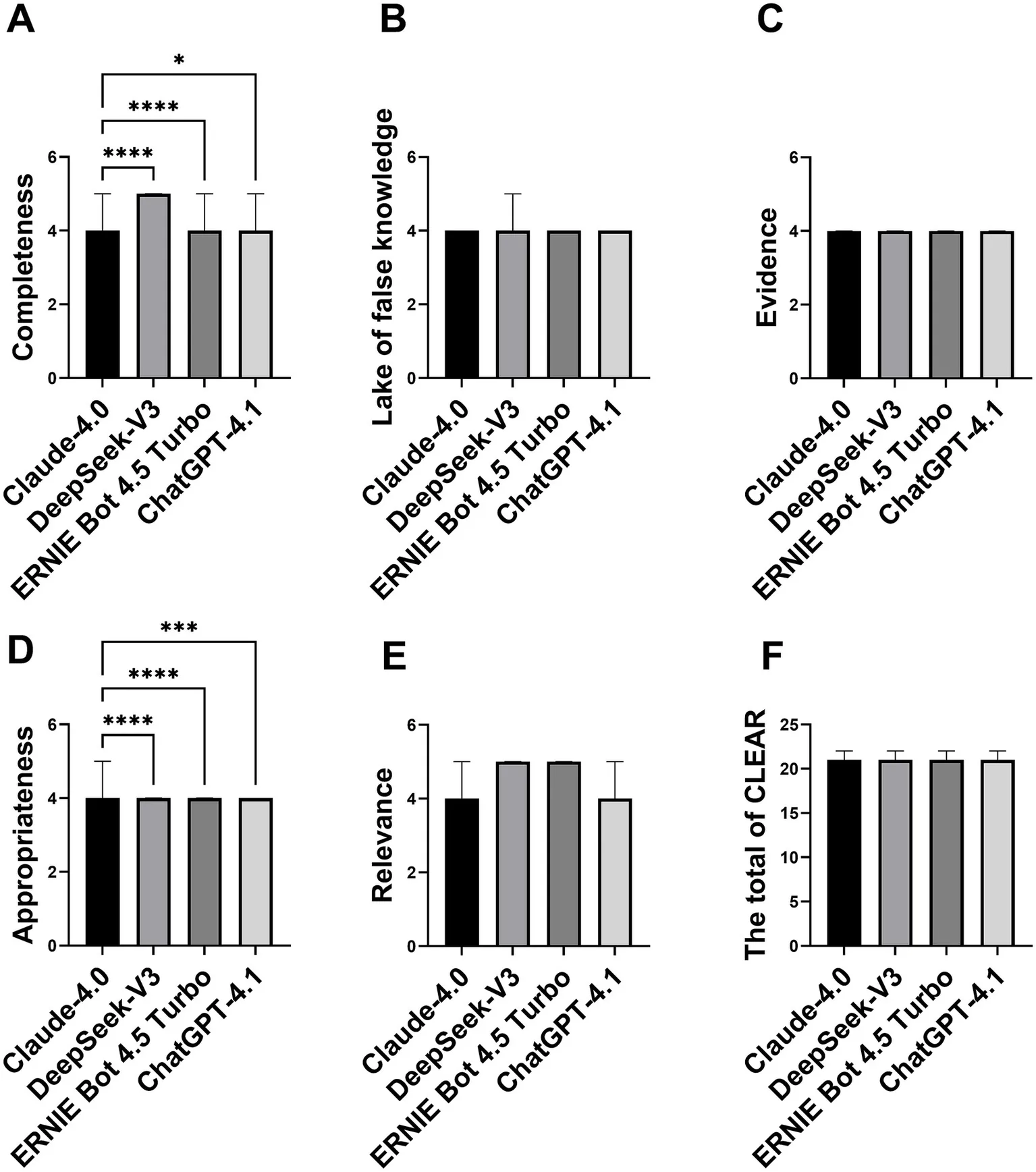
Bonferroni pairwise comparisons were performed among the four LLMs (Claude-4.0, DeepSeek-V3, ERNIE Bot 4.5 Turbo, and ChatGPT-4.1) across five CLEAR dimensions to identify specific differences: **(A)** completeness, **(B)** lack of false information, **(C)** evidence-based, **(D)** appropriateness, and **(E)** relevance. **(F)** The total of CLEAR. The difference between unmarked pairings was not statistically significant. *means *p* < 0.05, ****means *p* < 0.0001.

We evaluated the models using the CLEAR tool across five dimensions. The data were tested using the Kolmogorov–Smirnov test and were found not to follow a normal distribution. Detailed data is shown in Figure 2 and Table 4.

**Table 4:** The evaluation results of the four AI models on CLEAR and PEMAT-P.

Variable	Descriptive statistics	Claude-4.0 (*N* = 124)	DeepSeek-V3 (*N* = 124)	ERNIE Bot 4.5 Turbo (*N* = 124)	ChatGPT-4.1 (*N* = 124)	χ^2^	Kendall’s W	*P* value
CLEAR								
Completeness	Mean ± SD	3.96 ± 0.71	4.43 ± 0.67	4.40 ± 0.65	4.28 ± 0.67	47.661	0.128	<0.001
	Median (IQR)	4.00 (3.00–5.00)	5.00 (4.00–5.00)	4.00 (4.00–5.00)	4.00 (4.00–5.00)			
Lack of false information	Mean ± SD	4.04 ± 0.55	4.19 ± 0.54	4.10 ± 0.44	4.08 ± 0.49	7.644	0.021	0.054
	Median (IQR)	4.00 (4.00–4.00)	4.00 (4.00–5.00)	4.00 (4.00–4.00)	4.00 (4.00–4.00)			
Evidence	Mean ± SD	3.76 ± 0.55	3.77 ± 0.67	3.81 ± 0.56	3.84 ± 0.56	2.309	0.006	0.511
	Median (IQR)	4.00 (3.00–4.00)	4.00 (3.00–4.00)	4.00 (3.00–4.00)	4.00 (3.25–4.00)			
Appropriateness	Mean ± SD	4.26 ± 0.66	3.70 ± 0.53	3.67 ± 0.51	3.82 ± 0.46	88.360	0.238	<0.001
	Median (IQR)	4.00 (4.00–5.00)	4.00 (3.00–4.00)	4.00 (3.00–4.00)	4.00 (4.00–4.00)			
Relevance	Mean ± SD	4.48 ± 0.52	4.60 ± 0.49	4.52 ± 0.50	4.44 ± 0.53	7.516	0.020	0.057
	Median (IQR)	4.00 (4.00–5.00)	5.00 (4.00–5.00)	5.00 (4.00–5.00)	4.00 (4.00–5.00)			
The total of CLEAR	Mean ± SD	20.50 ± 2.28	20.69 ± 1.83	20.52 ± 1.67	20.47 ± 1.83	1.985	0.005	0.576
	Median (IQR)	21.00 (19.00–22.00)	21.00 (19.00–22.00)	21.00 (19.00–22.00)	21.00 (19.00–22.00)			
PEMAT-P								
Understandability	Mean ± SD	83.60 ± 7.09	79.84 ± 5.29	88.23 ± 4.62	92.81 ± 7.91	159.120	0.428	<0.001
	Median (IQR)	83.33 (76.70–90.91)	83.33 (75.00–83.33)	91.67 (83.33–91.67)	91.91 (91.91–100.00)			
Actionability	Mean ± SD	60.00 ± 0	60.22 ± 1.18	75.32 ± 1.23	60.40 ± 1.98	354.023	0.952	<0.001
	Median (IQR)	60.00 (60.00–60.00)	60.00 (60.00–60.00)	75.00 (75.00–75.00)	60.00 (60.00–60.00)			

N, sample size, SD, standard deviation, IQR, interquartile range, CLEAR, a 5-point Likert scale (Completeness, Lack of false information, Evidence, Appropriateness, Relevance), χ^2^, Friedman chi-square statistic, Kendall’s W, Kendall’s coefficient of concordance (effect size for the Friedman test), Kendall’s W values of 0.10, 0.30, and 0.50 were considered small, moderate, and large effects, respectively.

Overall, the four LLMs achieved high CLEAR scores across all five domains, with median scores generally ranging from 4 to 5, indicating good overall content quality. Differences among models were primarily observed in Completeness and Appropriateness, whereas performance was largely comparable for Lack of False Information, Evidence, and Relevance. The data revealed statistically significant differences in Completeness (χ^2^ = 47.661, *p* < 0.001, Kendall’s W = 0.128) and Appropriateness (χ^2^ = 88.360, *p* < 0.001, ε^2^ = 0.238). In contrast, the four AI models performed comparably in Lack of False Information (χ^2^ = 7.644, *p* = 0.054, Kendall’s W = 0.021), Evidence(χ^2^ = 2.309, *p* = 0.511, Kendall’s W = 0.006), and Relevance (χ^2^ = 7.516, *p =* 0.133, Kendall’s W = 0.020), with no statistically significant differences observed. The relatively small effect sizes in these domains indicated limited practical differences among the four LLMs. Claude-4.0 received the lowest completeness score (median 4.00, IQR 3.00–5.00), suggesting that its responses more frequently omitted clinically relevant information compared with the other models. Conversely, DeepSeek-V3 and ERNIE Bot 4.5 Turbo consistently achieved higher completeness scores, indicating that they generally provided more comprehensive responses. For Appropriateness, Claude-4.0 significantly outperformed the other three large AI models (median4.00, IQR 4.00–5.00). No significant differences were found among DeepSeek-V3, ERNIE Bot 4.5 Turbo, and ChatGPT-4.1. The inter-rater reliability for the overall CLEAR score was excellent, with an ICC(2,k) of 0.92 (95% CI: 0.90–0.94).

### 3.3 PEMAT-P assessment

**Figure 3:**
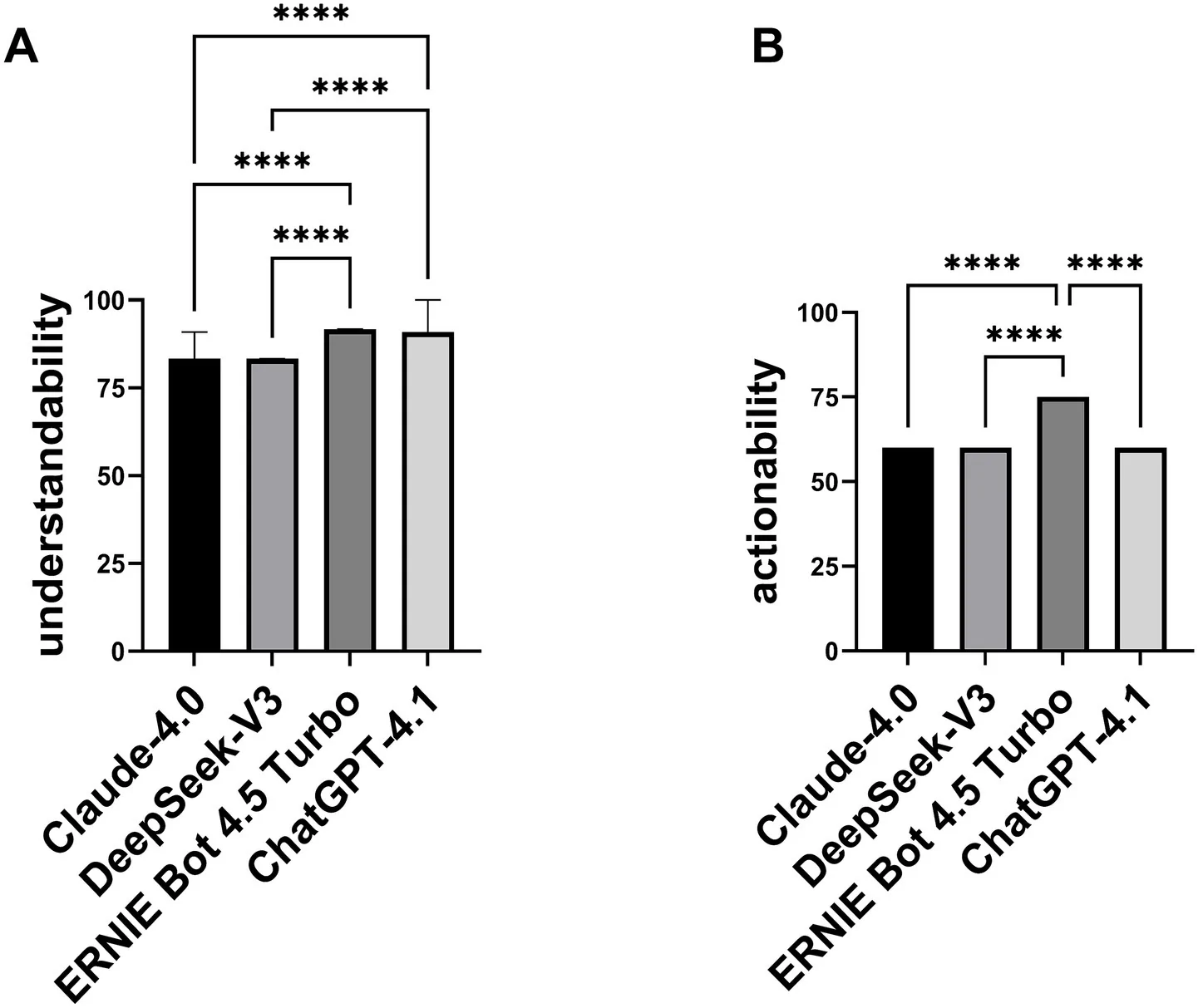
Comparison of four LLMs (Claude-4.0, DeepSeek-V3, ERNIE Bot 4.5 Turbo, and ChatGPT-4.1) on the PEMAT-P scale evaluation: results for **(A)** understandability, **(B)** actionability. The difference between unmarked pairings was not statistically significant. ****means *p* < 0.0001.

We use the Patient Education Materials Assessment Tool (PEMAT-P) to evaluate understandability and actionability. The data were assessed for normality using the Kolmogorov–Smirnov test and were found to deviate from a normal distribution. Both understandability (χ^2^ = 159.120, *p* < 0.001, Kendall’s W = 0.428) and actionability (χ^2^ = 354.023, *p* < 0.001, Kendall’s W = 0.952) showed significant differences. The effect size analysis demonstrated large effects for both PEMAT-P domains, indicating substantial differences among the four LLMs in terms of understandability and actionability. Table 4 and Figure 3 present the evaluation results.

For understandability, ChatGPT-4.1 achieved the highest score (median 91.91, IQR 91.91–100.00), followed by ERNIE Bot 4.5 Turbo (median 91.67%, IQR 83.33–91.67%), Claude-4.0 (median 83.33%, IQR 76.70–90.91%), and DeepSeek-V3 (median 83.33%, IQR 75.00–83.33%). All four AI models scored above 70%, demonstrating good understandability. In contrast to understandability, actionability varied considerably across models. For actionability, ERNIE Bot 4.5 Turbo achieved the highest (median 75.00%, IQR 75.00–75.00%), ChatGPT-4.1 (median 60.00%, IQR 60.00–60.00%), DeepSeek-V3 (median 60.00%, IQR 60.00–60.00%), and Claude-4.0(median 60.00%, IQR 60.00–60.00%). No significant differences were observed among ChatGPT-4.1, DeepSeek-V3, and Claude-4.0. ERNIE Bot 4.5 Turbo achieved the highest actionability score (median 75.00%), whereas the remaining three models all showed median scores of 60.00%, indicating that their responses generally contained fewer specific and actionable recommendations for patients. The inter-rater reliability for PEMAT-P was good to excellent, with ICC(2,k) values of 0.89 (95% CI: 0.87–0.91) for understandability and 0.86 (95% CI: 0.83–0.89) for actionability.

## 4 Discussion

### 4.1 Principal findings

In this study, we conducted a statistical test on the content quality and readability of the responses generated by Claude-4.0, ChatGPT-4.1, DeepSeek-V3 and ERNIE Bot 4.5 turbo on type 2 diabetes. We find that the four models are similar in the overall level of information quality, but different in specific dimensions. Although the appropriateness of Claude-4.0 is better than the other three models, it is the lowest among the four models in terms of integrity. In terms of patient education, ChatGPT-4.1 exhibited the highest understandability. Interestingly, in actionability, ERNIE Bot 4.5 turbo is obviously superior to the other three large models, showing the characteristics of easy operation, while the other large models do not perform well in this dimension.

### 4.2 Content quality

In terms of content quality, the four AI models are similar in the three core content quality dimensions of Lack of false information, Evidence and Relevance. This finding shows that after rapid iteration in recent years, the mainstream large language model has developed to a similar level in the factual accuracy, evidence-based support and topic relevance of medical health information. This contrasts with the uneven performance of different models reported in earlier studies, reflecting the overall improvement of the maturity of the application of large models in the medical field (33, 34). It is worth noting that Claude is better than the other three LLMs in terms of Appropriateness, but lower than the other three models in terms of Completeness. It was also found in another study on preoperative patient education for superior capsular reconstruction that the Claude score was low in the Completeness dimension (35). This may be because Claude prefers to output focused and concise information when generating answers, which leads to the performance that his answers are not deep enough and have low integrity. This feature is well explained by the fact that the number of characters generated by Claude-4.0 is less than half of that of ERNIE Bot 4.5 turbo. The results of Appropriateness were similar to the comparative study of preoperative patient education materials, and Claude also performed best. However, this study found that Claude performed equally well in the completeness dimension, which was different from that of other models in this study (36). These different research results suggest that the different performance of Claude in the dimensions of Completeness and Appropriateness may be related to the evaluation object, the evaluation scenario and the version of Claude used, which still needs to be further verified in larger samples and multiple scenarios. In addition, we found that the four AI models scored consistently low in the dimension of Evidence. This result may be due to the lack of active annotation mechanism for evidence sources when LLMs generates content. Another possible reason is that this study does not explicitly require the model to quote literature or give evidence in the prompt word. However, it is difficult to steadily improve this problem even through the guidance of prompt words. On the one hand, previous studies have shown that prompt words are not a reliable evidence-based mechanism, and the same type of prompt words show significant differences in different models, or even different versions of the same model (37). On the other hand, LLMs tends to generate fictitious or erroneous references when it is explicitly required to provide evidence (38). Therefore, the relatively low evidence-support scores are more likely to reflect an inherent limitation of LLMs in factual attribution, rather than being attributable solely to prompt design. Enhancing the reliability of LLMs in evidence-based contexts should no longer rest on the prompts supplied by users. Considering this, we recommend that developers integrate trusted medical databases into the model so that the generation of medical content can be grounded in the latest scientific advances, and that outputs be accompanied by traceable citation links, thereby reducing the risk of fabricating references.

### 4.3 Readability

The understandability of all LLMs exceeded the threshold of 70%, indicating that the generated health information is generally understandable to ordinary readers. ChatGPT-4.1 is the most understandable. ChatGPT has achieved high understandable scores in the research of patellar tendinopathy and cardiac catheterization (21, 39). This evidence shows that ChatGPT has significant advantages in generating patient-friendly and easy to understand health information. At the same time, it is consistent with its high character generation rate and simplified response architecture, which may help to generate clear and concise content. The low understandability of DeepSeek-V3 may reflect its tendency to generate more intensive and more technical responses, which is consistent with its long response time and medium number of characters. It is noteworthy that regarding actionability, only ERNIE Bot 4.5 turbo has attained a threshold of 70%, whereas the other three models are not user-friendly. In a study on patient education texts for self-management of chronic heart failure (CHF), ERNIE Bot 4.5 Turbo achieved the highest operability among all adopted models (40). The poor performance of claude-4.0, chatgpt-4.1 and deepseek-v3 in this dimension shows that although the information generated by them performs well in comprehensibility, these models do not give clear operation steps and instructions and cannot provide sufficient operable behavior guidance for patients. The advantages of ERNIE Bot 4.5 turbo in operability may be attributed to its training corpus and the optimization of the educational environment for Chinese patients. In this environment, specific and practical guidance is an element commonly expected in culture in health information. These findings indicate that the future optimization of large language models for patient education applications should focus on generating operable and step-by-step guidance content, not just general information content.

### 4.4 Limitations

This study has several limitations that need to be acknowledged. Firstly, although the Chinese popular science guide for type 2 diabetes represents an authoritative and clinically validated reference standard, its focus on the Chinese population may limit the applicability of the research results in other language and cultural backgrounds. Secondly, this study only evaluates the performance of large-scale language model in the single round and context free query format, which is not completely consistent with the real situation of ordinary users using large-scale language model. Thirdly, this study presents the performance of the models based on a single day’s data and does not measure the same tasks at different time points. Consequently, it fails to capture the dynamic changes in the performance of large language models. In addition, response time and time to first byte were measured through the browser interface. The recorded values may be subject to a certain degree of measurement error. However, this measurement approach better reflects the response speed perceived by users during real-world interactions. Therefore, our findings are more appropriate for evaluating product-level user experience rather than providing a strict comparison of model inference performance.

Therefore, future research should evaluate the health education capabilities of large language models across multiple languages, further optimize context integration and personalized response generation, and provide more targeted health recommendations. Future studies should also incorporate repeated measurements to better assess the stability of LLM performance over time.

## 5 Conclusion

Our research shows that the four models have tended to be consistent in the core content quality dimensions of accuracy, evidence support and relevance. However, the differences between the models are more reflected in the way of information presentation and the practicality of patients. Claude-4.0 is outstanding in suitability but relatively insufficient in completeness. ChatGPT-4.1 has significant advantages in comprehensibility, while ERNIE Bot 4.5 turbo is the only model that reaches the practical threshold in operability. Clinicians and health informatics experts who integrate the big language model into diabetes education or self-management support projects should fully understand the capabilities of different models, avoid excessive dependence on a single model, and formulate differentiated deployment strategies according to specific application scenarios. Future research should further expand the multilingual, multicultural and multi round dialogue framework to effectively enhance its credibility and clinical application value in professional medical and health scenes.

## Data Availability

The original contributions presented in the study are included in the article/Supplementary material, further inquiries can be directed to the corresponding author/s.
