# Supplementary material for: Evaluating large language models using the Type 2 Diabetes Health Education guideline: a comparative analysis of ChatGPT-4.1, Claude-4.0, DeepSeek-V3, and ERNIE Bot 4.5 Turbo
Source: Front Public Health. 2026 Aug 19;14:1900281. doi: 10.3389/fpubh.2026.1900281 (PMC13537033; doi:10.3389/fpubh.2026.1900281)
Supplement: Supplementary file 1 [file Table_1.DOCX]

Supplementary Material

**Supplementary Table 1.** Complete list of the 124 prompts used for LLM evaluation, consisting of the original questions from the "Chinese Type 2 Diabetes Popular Science Guidelines" (《中国2型糖尿病科普版指南》)

| Number | Questions |
| --- | --- |
| Chapter 1. Diagnosis and Classification of Diabetes | |
| 1 | What is blood glucose? |
| 2 | What is the normal blood glucose concentration in a healthy person? |
| 3 | What is diabetes? |
| 4 | What are the typical symptoms of diabetes? |
| 5 | How is diabetes diagnosed? |
| 6 | What are the different types of diabetes? |
| Chapter 2. Diabetes Screening and Assessment | |
| 1 | How can individuals at high risk for diabetes be identified? |
| 2 | How is diabetes screened for? |
| 3 | Why is a comprehensive evaluation necessary at the time of an initial diabetes diagnosis? |
| 4 | After being diagnosed with diabetes, how can patients modify unhealthy lifestyle habits and comprehensively manage their diabetes under the guidance of a specialist? |
| 5 | What information should patients provide to their physician during a medical consultation? |
| 6 | How often should patients attend follow-up visits, and what examinations should be performed during follow-up? |
| Chapter 3. Diabetes Self-Management Education and Support | |
| 1 | Why do people with diabetes need to learn about diabetes? |
| 2 | What are the goals of diabetes education and management? |
| 3 | What are the ways for people with diabetes to acquire knowledge about diabetes? |
| 4 | What knowledge should people with diabetes learn and master? |
| 5 | At which key stages should people with diabetes receive diabetes education or refresh their knowledge? |
| 6 | Why is self-monitoring of blood glucose important for people with diabetes? |
| 7 | How should self-monitoring of blood glucose be performed? |
| 8 | At what time points should self-monitoring of blood glucose be performed? |
| 9 | Why should glycated hemoglobin be monitored regularly? |
| 10 | How should other risk factors be monitored? |
| 11 | What should people with diabetes do if they lose interest in daily activities, become irritable, or experience insomnia? |
| 12 | What new technologies can people with diabetes use to learn about diabetes? |
| Chapter 4. Comprehensive Management Targets for Type 2 Diabetes | |
| 1 | What treatment targets should be controlled in people with type 2 diabetes? |
| 2 | Why is it important for people with diabetes to control their blood pressure and blood lipid levels? |
| 3 | What is individualized glycemic target setting? |
| 4 | Why can blood glucose levels still fluctuate considerably even when HbA1c is within the target range? |
| 5 | What are the potential harms of blood glucose fluctuations? |
| 6 | Under what circumstances should the glucose-lowering treatment regimen be adjusted? |
| 7 | What is the simplified treatment pathway for hyperglycemia in type 2 diabetes? |
| Chapter 5. Medical Nutrition Therapy for Diabetes | |
| 1 | What are the goals of medical nutrition therapy for diabetes? |
| 2 | What are the principles of medical nutrition therapy for diabetes? |
| 3 | How should the three major macronutrients be distributed in medical nutrition therapy for diabetes? |
| 4 | What other nutrients should be considered in medical nutrition therapy for diabetes? |
| 5 | What dietary patterns can help achieve weight loss in medical nutrition therapy for diabetes? |
| 6 | What is a low-carbohydrate diet? |
| 7 | What is a high-protein diet? |
| 8 | What is a low glycemic index diet? |
| 9 | Can people with diabetes drink alcohol? |
| 10 | What dietary precautions should women with gestational diabetes mellitus take? |
| Chapter 6. Exercise Therapy for Diabetes | |
| 1 | What are the benefits of exercise for people with diabetes? |
| 2 | What principles should people with diabetes follow when exercising? |
| 3 | Which type of exercise is most effective for weight loss in exercise therapy for diabetes? |
| 4 | What preparations should people with diabetes make before exercising? |
| 5 | What should people with diabetes pay attention to after exercising? |
| 6 | How can people with diabetes evaluate whether their exercise intensity is appropriate during exercise therapy? |
| 7 | Under what circumstances should people with diabetes avoid exercise? |
| Chapter 7. Pharmacological Treatment of Hyperglycemia | |
| 1 | What are the major classes of oral glucose-lowering medications? |
| 2 | What are the characteristics of metformin? |
| 3 | What are the characteristics of sulfonylureas? |
| 4 | What are the characteristics of meglitinides? |
| 5 | What are the characteristics of thiazolidinediones? |
| 6 | What are the characteristics of α-glucosidase inhibitors? |
| 7 | What are the characteristics of DPP-4 inhibitors? |
| 8 | What are the characteristics of SGLT2 inhibitors? |
| 9 | What are the major types of insulin? |
| 10 | How can the correct timing of insulin injection be determined? |
| 11 | Under what circumstances should insulin therapy be initiated? |
| 12 | How should insulin doses be adjusted? |
| 13 | What is short-term intensive insulin therapy? |
| 14 | What are the characteristics of GLP-1 receptor agonists? |
| 15 | What types of GLP-1 receptor agonists are available? |
| 16 | What are the advantages of combining GLP-1 receptor agonists with basal insulin? |
| 17 | Which glucose-lowering medications have cardiovascular or renal protective effects? |
| 18 | How can gastrointestinal adverse reactions caused by glucose-lowering medications be reduced? |
| Chapter 8. Weight Management for People with Type 2 Diabetes | |
| 1 | What are the benefits of weight loss for people with type 2 diabetes? |
| 2 | How should weight loss goals be appropriately set for people with type 2 diabetes? |
| 3 | What does lifestyle intervention for weight loss include in people with type 2 diabetes? |
| 4 | Which glucose-lowering medication promote weight loss? |
| 5 | Can metabolic surgery be used to treat diabetes? |
| Chapter 9. Diabetes-Related Technologies | |
| 1 | What blood glucose monitoring methods are commonly used in clinical practice, and what are their advantages and disadvantages? |
| 2 | What is continuous glucose monitoring? |
| 3 | Who are suitable candidates for continuous glucose monitoring? |
| 4 | What is time in range? |
| 5 | What insulin delivery devices are available? |
| 6 | What are the characteristics of insulin syringes? |
| 7 | What is an insulin pen? |
| 8 | How should insulin pen needles be selected? |
| 9 | What precautions should be taken when injecting insulin? |
| 10 | How should insulin leakage after injection be managed? |
| 11 | How can injection pain be reduced when using diabetes-related technologies? |
| 12 | What is a needle-free injector? |
| 13 | What is insulin pump therapy? |
| 14 | Who are suitable candidates for insulin pump therapy? |
| Chapter 10. Hypoglycemia | |
| 1 | What is hypoglycemia, and what are its potential harms? |
| 2 | What are the symptoms of hypoglycemia? |
| 3 | How is hypoglycemia classified? |
| 4 | What are the causes of hypoglycemia? |
| 5 | How should hypoglycemia be managed if symptoms occur? |
| 6 | How can hypoglycemia be prevented? |
| 7 | What is the Somogyi effect? |
| Chapter 11. Acute Complications of Diabetes | |
| 1 | What is diabetic ketoacidosis? |
| 2 | What are the causes of diabetic ketoacidosis? |
| 3 | What are the symptoms of diabetic ketoacidosis? |
| 4 | What is the hyperosmolar hyperglycemic state? |
| 5 | What are the clinical manifestations of the hyperosmolar hyperglycemic state? |
| Chapter 12. Chronic Complications of Diabetes | |
| 1 | What are the common chronic complications of diabetes? |
| 2 | What is diabetic kidney disease? |
| 3 | How is diabetic kidney disease screened for? |
| 4 | What are the stages of diabetic kidney disease? |
| 5 | What dietary considerations are important for people with diabetic kidney disease? |
| 6 | How should glucose-lowering medications be selected for people with diabetic kidney disease? |
| 7 | How should blood pressure be managed in people with diabetic kidney disease? |
| 8 | How is diabetic retinopathy screened for? |
| 9 | How is diabetic neuropathy screened for? |
| 10 | How is lower extremity atherosclerotic disease screened for in people with diabetes? |
| 11 | What is diabetic foot, and what are its potential harms? |
| 12 | How is diabetic foot screened for? |
| 13 | How can diabetic foot be prevented? |
| Chapter 13. Cardiovascular Disease and Risk Factor Management | |
| 1 | What is cardiovascular disease? |
| 2 | What is atherosclerotic cardiovascular disease? |
| 3 | Why should people with diabetes pay attention to cardiovascular disease? |
| 4 | Which cardiovascular risk factors should be screened for in people with diabetes? |
| 5 | How should cardiovascular risk be assessed? |
| 6 | How can people with diabetes manage cardiovascular risk factors? |
| Chapter 14. Special Populations with Type 2 Diabetes | |
| 1 | What is diabetes during pregnancy? |
| 2 | What are the potential risks of hyperglycemia during pregnancy for both the mother and the baby? |
| 3 | What dietary principles should women with gestational diabetes mellitus follow? |
| 4 | Which glucose-lowering medications are recommended during pregnancy for women with gestational diabetes mellitus? |
| 5 | What are the characteristics of type 2 diabetes in children and adolescents? |
| 6 | Which glucose-lowering medications can be used for children and adolescents with type 2 diabetes? |
| 7 | What are the characteristics of diabetes in older adults? |
| 8 | What should be considered when selecting glucose-lowering medications for older adults with diabetes? |
